# Defining prognosis in sarcoidosis

**DOI:** 10.1097/MD.0000000000023100

**Published:** 2020-11-25

**Authors:** Mariana Carneiro Lopes, Thaís Porto Amadeu, Marcelo Ribeiro-Alves, Claudia Henrique da Costa, Bruno Rangel Antunes Silva, Luciana Silva Rodrigues, Elisabeth Jauhar Cardoso Bessa, Leonardo Palermo Bruno, Agnaldo José Lopes, Rogerio Rufino

**Affiliations:** a Department of Thoracic Disease; b Department of Pathology and Laboratories, State University of Rio de Janeiro; c National Institute of Infectology Evandro Chagas, Oswaldo Cruz Foundation, Rio de Janeiro, Brazil.

**Keywords:** prognosis, sarcoidosis, score-based predictive model

## Abstract

Sarcoidosis is a multi-systemic granulomatous disease. Affected individuals can show spontaneous healing, develop remission with drug treatment within 2 years, or become chronically ill. Our main goal was to identify features that are related to prognosis.

The study consisted of 101 patients, recruited at a single center, who were already diagnosed with sarcoidosis at the start of the study or were diagnosed within 48 months. Ninety individuals were followed-up for at least 24 months and were classified according to clinical outcome status (COS 1 to 9). Those with COS 1–4 and COS 5–9 were classified as having favorable and unfavorable outcomes, respectively. Unconditional logistic regression analyses were conducted to define which variables were associated with sarcoidosis outcomes. Subsequently, we established a scoring system to help predict the likelihood of a favorable or unfavorable outcome.

Of our patients, 48% developed a chronic form of the disease (COS 5–9). Three clinical features were predictive of prognosis in sarcoidosis. We built a score-based model where the absence of rheumatological markers (1 point), normal pulmonary functions (2 points), and the presence of early respiratory symptoms manifestations (2 points) were associated with a favorable prognosis. We predicted that a patient with a score of 5 had an 86% (95% confidence interval [CI] 74%–98%) probability of having a favorable prognosis, while those with scores of 4, 3, 2, 1, and 0 had probabilities of 72% (95% CI 59–85%), 52% (95% CI 40–63%), 31% (95% CI 17–44%), 15% (95% CI 2–28%), and 7% (95% CI 0–16%) of having a favorable prognosis, respectively. Thus, our easy-to-compute algorithm can help to predict prognosis of sarcoidosis patients, facilitating their management.

## 1 Introduction

Sarcoidosis is a multi-systemic granulomatous disease whose origin is not yet well established. The sarcoidosis incidence among populations globally varies between 1 and 120 cases per 100,000 people, with the highest and lowest incidences registered among African-Americans and Japanese, respectively.^[1–3]^ In Brazil, the estimated incidence is below 10 per 100,000 people.^[4]^ Sarcoidosis affects both men and women with a similar frequency, with the main onset occurring between 25 and 45 years of age, and in some regions, with a second onset window between 50 and 60 years of age.^[5–9]^

There is still no well-defined etiology for sarcoidosis. There is some growing evidence that the sarcoidosis-related immune response may also include an autoimmune response with some reaction to the bodys own proteins. However, sarcoidosis is not considered to be a classic autoimmune disease, such as rheumatoid arthritis or systemic lupus erythematosus.

It appears that up to 10% of affected cases can be attributed to some familial predisposition.^[10]^ Some human leukocyte antigen (HLA) class 2 genes have already been identified as being associated with genetic susceptibility, and patients may manifest the disease after some environmental exposure-, occupational-, or infection-related triggers.^[11,12]^

The course of the disease is quite variable. It can remit spontaneously or with treatment within 2 years, but some patients can progress to a chronic form, requiring treatment including corticosteroid therapy for a long time.^[1]^ Mortality is usually around 1% to 5%.^[13]^ Some features that have already been studied, such as erythema nodosum, acute arthritis, and bilateral hilar lymph node enlargement, are related to a better prognosis. However, cardiac and neurological sarcoidosis, lupus pernio, forced vital capacity <80%, fibrosis, and pulmonary hypertension are related to the worst prognosis.^[14–16]^

The main goal of this study was to identify features that are related to prognosis of this disease, and to establish a scoring system that can predict the clinical progression of patients with sarcoidosis.

## 2 Methods

### 2.1 Study design and population

The relevant research ethics committee approved this study (protocol No. 1158044). For the study, a cohort of 101 patients was recruited at a single center. Patients were already diagnosed with sarcoidosis at the study inception or were diagnosed within 48 months, with a disease duration ranging from 2 months to 30 years. Sarcoidosis diagnosis was determined according to the American Thoracic Society (ATS), European Respiratory Society (ERS), and World Association of Sarcoidosis and Other Granulomatous Disorders (WASOG) guidelines on sarcoidosis.

### 2.2 Data collection

During follow-up, we collected data on the clinical features, pulmonary functional tests, computed tomographic findings, pulmonary artery pressure (estimated by echocardiography), laboratory tests (antinuclear antibodies, rheumatoid factor, and anti-neutrophil cytoplasmic antibodies), early clinical manifestations, and patients evolution according to treatment. The respiratory clinical manifestations included chest pain, dyspnea, cough, and wheezing. The constitutional symptoms were fever, weight loss, fatigue, arthralgia, and night sweats. Palpitations and angina were listed as cardiac symptoms. Facial palsy and balance disturbance were considered as neurological symptoms.

Radiological patterns were classified as defined by scadding,^[17]^ and also according to the computed tomographic findings of ground-glass infiltrate, consolidations, mediastinal and hilar lymph node enlargement, micronodular infiltrates and nodular opacities, traction bronchiectasis, honeycomb and fibrosis, septal thickening, and peribronchovascular thickening.^[18]^ Transthoracic echocardiography was used to estimate pulmonary artery systolic pressure (PASP). PASP >35 mm Hg was considered abnormal.^[19]^

Pulmonary functional tests were classified as abnormal when at least one of the following criteria were met: forced expiratory volume in 1 second (FEV_1_) <80%, forced vital capacity (FVC) <80%, and FEV_1_/FVC <70. Rheumatological markers were considered present if antinuclear antibodies (ANA) titers were ≥1:160 and if either rheumatoid factor (RF) or anti-neutrophil cytoplasmic antibodies (ANCA) were detected in blood samples.^[1]^

We used the clinical outcome status (COS) criterion defined by Baughman et al^[20]^ to classify the evolution of patients.

### 2.3 Statistical analysis

Sample calculation revealed that at least 110 patients were required to obtain a 90% confidence level with a standard deviation of 10 and a confidence interval width (2-sided) of 3.

To describe the sociodemographic and clinical characteristics of the study population classified as having either unfavorable, undefined, or favorable clinical evolution, non-parametric Kruskal–Wallis tests were used for continuous variables, and Fishers exact tests were used for comparison of relative frequencies of categorical variables.

The protection/risk estimation for a favorable clinical evolution as compared to an unfavorable evolution, was calculated as an adjusted odds ratio (aOR) and 95% confidence interval (95% CI) for each variable, using unconditional logistic regression models. To account for selection biases, sociodemographic, clinical, and laboratory characteristics associated with the outcome of interest at *P* values <.2 in the bivariate analysis were included as confounders in multiple unconditional logistic regression models. We developed a scoring system for risk stratification using adjusted parameters of the multiple unconditional logistic regression models with all variables associated with a favorable clinical evolution.^[21]^

All statistical analyses were performed using R version 3.6.1 (R Core Team, 2019).

## 3 Results

### 3.1 Patient characteristics

One hundred five patients from the State University of Rio de Janeiro (Brazil) diagnosed with sarcoidosis were followed-up between July 2015 and July 2019. Four patients were lost to follow-up, and 11 patients had less than 24 months of follow-up. Ninety individuals were followed-up for at least 24 months, and were classified according to clinical outcome status (COS 1–9) (Table 1). Those with COS 1–4 and COS 5–9 were classified as having favorable and unfavorable outcomes, respectively, while the 11 patients with less than 24 months of follow-up were classified as having undetermined outcomes.

**Table 1:** Clinical outcome status (COS) classification.

COS	Definition	n = 90 (%)
1	Disease resolved, never treated	7 (7)
2	Disease resolved, without treatment for more than 1 year	23 (25)
3	Minimal disease^∗^, never treated	1 (1)
4	Minimal disease, untreated for more than one year	15 (16)
5	Persistent disease, never treated	3 (3)
6	Persistent disease, untreated for more than 1 year	3 (3)
7	In treatment, without worsening in the last year, asymptomatic	16 (17)
8	In treatment, without worsening in the last year, symptomatic	8 (8)
9	In treatment, worsening^†^ in the last year	14 (15)

We describe the epidemiological, functional, and radiological characteristics of these 101 individuals in Table 2. The mean age at diagnosis was 44 years. Only 35% of patients were Caucasians. Patients were predominantly women (68%) and were not current or past smokers (72%). Radiological stage 2 was the most prevalent, and twenty one patients (23%) had some positive rheumatological markers (ANA, RF, or ANCA). Moreover, 49% of the patients had some abnormal pulmonary function test results (FEV_1_ <80% or FVC <80%, or FEV_1_/FVC <70), with only 3 patients with estimated PASP >35 mm Hg. The main initial clinical manifestations were respiratory symptoms and skin lesions, as illustrated in Table 3.

**Table 2:** Characteristics of 101 patients diagnosed with sarcoidosis.

Feature	OverallN = 101	Unfavourable clinical evolutionN = 44	Undefined clinical EvolutionN = 11	Favourable clinical EvolutionN = 46	*P* value
Age(years)	53 (IQR = 17)	51.5 (IQR = 18.25)	50 (IQR = 12.5)	55 (IQR = 19.75)	.16
Race (%)					
Non-Caucasian	66 (65.3)	28 (27.7)	6 (5.9)	32 (31.7)	.61
Caucasian	35 (34.7)	16 (15.8)	5 (5)	14 (13.9)	
Sex					
female	69 (68.3)	32 (31.7)	6 (5.9)	31 (30.7)	.45
male	32 (31.7)	12 (11.9)	5 (5)	15 (14.9)	
Smoking (current or past)					
no	72 (72.7)	33 (32.7)	8 (7.9)	31 (30.7)	.73
yes	27 (27.3)	10 (9.9)	3 (3)	14 (13.9)	
Diagnostic Age (years)	44 (IQR = 17)	44.5 (IQR = 16.25)	50 (IQR = 13)	41.5 (IQR = 17.75)	.42
Radiological Staging (%)					
1	14 (13.9)	4 (4)	0 (0)	10 (9.9)	.26
2	52 (51.5)	25 (24.8)	7 (6.9)	20 (19.8)	
3	17 (16.8)	6 (5.9)	1 (1)	10 (9.9)	
4	18 (17.8)	9 (8.9)	3 (3)	6 (5.9)	
Rheumatological markers - ANA, RF or ANCA (%)					
negative	70 (76.9)	27 (26.7)	9 (8.9)	34 (33.7)	.21
positive	21 (23.1)	13 (12.9)	1 (1)	7 (6.9)	
Pulmonary arterial hypertension					
no	92 (96.8)	39 (38.6)	10 (9.9)	43 (42.6)	.72
yes	3 (3.2)	2 (2)	0 (0)	1 (1)	
FEV_1_ (%)^∗^	83 (IQR = 23.5)	75 (IQR = 25.5)	84 (IQR = 24)	86 (IQR = 15.75)	.002
FEV_1_/FVC (%)^∗^	80.5 (IQR = 11)	80 (IQR = 10)	82 (IQR = 6.5)	80 (IQR = 11.75)	.82
FVC (%)^∗^	83.5 (IQR = 24.25)	79 (IQR = 20.5)	81 (IQR = 16)	93.5 (IQR = 19.5)	.001
Pulmonary tests^∗^					
within the predicted	51 (51)	15 (14.9)	5 (5)	31 (30.7)	.007
Impaired	49 (49)	28 (27.7)	6 (5.9)	15 (14.9)	

**Table 3:** Clinical manifestations reported as initial symptoms by patients.

Early clinical manifestations	N (%)
Respiratory^∗^	62 (61)
Skin	46 (45)
Constitutional symptoms^†^	23 (22)
Asymptomatic	11 (10)
Ophthalmologic	10 (9)
Cardiology^‡^	6 (6)
Peripheral lymph node enlargement	3 (3)
Parotid gland	2 (2)
Neurology^§^	2 (2)
Queilitis	1 (1)
Nephrotic syndrome	1 (1)

The most prevalent computed tomographic findings (Table 4) were mediastinal lymph node enlargement (72%), and micronodular infiltrates and/or nodular opacities (59%).

**Table 4:** Prevalence of tomographic patterns in sarcoidosis patients.

Tomographic patterns	N (%)
Lymph node enlargement	72 (71)
Micronodular infiltrate and /or nodular opacities	59 (59)
Septal thickening	32 (32)
Ground glass	25 (25)
Traction bronchiectasis and/or honeycombing and/or fibrosis	17 (17)
Peribronchovascular Thickening	15 (15)
Consolidation	12 (12)

Ninety patients were followed for at least 24 months, and 48% developed a chronic form of the disease (COS 5, 6, 7, 8, or 9).

Unconditional logistic analyses were performed to define which variables were related to the more favorable outcome (Table 5). For these analyses, only the 90 patients with at least 24 months of follow-up were included.

**Table 5:** Variables related to the outcome in sarcoidosis.

Feature	Unfavourable evolution	Favourable evolution	Adjusted Model	*P* value
Race				
Non-Caucasian	28 (63.64)	32 (69.57)	0.93 (0.33–2.62)	.88
Caucasian	16 (36.36)	14 (30.43)		
Sex				
female	32 (72.73)	31 (67.39)	0.95 (0.33–2.7)	.92
male	12 (27.27)	15 (32.61)		
Smoking				
no	33 (76.74)	31 (68.89)	1.91 (0.6–6.05)	.27
yes		14 (31.11)		
Radiological stage				
2	10 (23.26)	20 (43.48)	3.39 (0.66–17.42)	.14
1	4 (9.09)	10 (21.74)		
3	6 (13.64)	10 (21.74)	1.54 (0.42–5.66)	.51
4	9 (20.45)	6 (13.04)	0.65 (0.18–2.36)	.51
Rheumatologic markers				
negative	27 (67.5)	34 (82.93)	0.19 (0.05–0.76)	.01
positive	13 (32.5)	7 (17.07)		
Pulmonary tests				
impaired	28 (65.12)	15 (32.61)	4.42 (1.59–12.3)	.004
normal	15 (34.88)	31 (67.39)		
Pulmonary arterial hypertension				
no	39 (95.12)	43 (97.73)	0.4 (0.03–5.56)	.49
yes	2 (4.88)	1 (2.27)		
Initial respiratory clinical manifestations				
yes	23 (52.27)	34 (73.91)	0.22 (0.07–0.65)	.006
no	21 (47.73)	12 (26.09)		

We then developed a scoring system to help predict the likelihood of a patient having a favorable or unfavorable outcome. Of our patients, 48% developed a chronic form of the disease (COS 5–9). Three clinical features were predictive of prognosis in sarcoidosis. We built a score-based model where the absence of rheumatological markers (1 point), normal pulmonary functions (2 points), and the presence of early respiratory symptoms manifestations (2 points) were associated with favorable prognosis. We predicted that a patient with a score of 5 would have an 86% (95% CI 74–98%) probability of having a favorable prognosis, while those with scores of 4, 3, 2, 1, and 0 had probabilities of 72% (95% CI 59–85%), 52% (95% CI 40–63%), 31% (95% CI 17–44%), 15% (95% CI 2–28%), and 7% (95% CI 0–16%) of having a favorable prognosis, respectively (Table 6).

**Table 6:** Probability score of favourable outcomes in sarcoidosis.

Score	Probability of favourable outcomes (CI 95%)
0	0.07 (−0.01–0.16)
1	0.15 (0.02–0.28)
2	0.31 (0.17–0.44)
3	0.52 (0.40–0.63)
4	0.72 (0.59–0.85)
5	0.86 (0.74–0.98)

## 4 Discussion

Sarcoidosis is a disease with variable presentation and course. Several characteristics that influence the patients prognosis have already been identified. In addition to describing the clinical, epidemiological, functional, and radiological characteristics of the 101 patients in our study, we aimed to establish a scoring system based on easily identifiable factors that could predict the clinical evolution of patients and help in the management of each case.

The mean age at diagnosis of our patients was 44 years, similar to that previously published, although we did not find a relationship between age and prognosis.^[22]^ Smoking was identified as a protective factor against sarcoidosis in other studies, but this was not confirmed by our results.^[23,24]^

Moreover, Kobak et al.^[25]^ showed the presence of rheumatological markers in sarcoidosis, with positive ANA testing in 28% and RF in 16% of patients. We observed that 23% of our patients tested positive for one of the rheumatological markers.

In 3 large study cohorts (ACCESS, MUSC, and TTS), the lung was the organ most affected by sarcoidosis (95%, 89%, and 99%, respectively), followed by the skin, eyes, and peripheral lymph nodes.^[21,26–28]^ All our patients had pulmonary involvement, although only 61% had respiratory symptoms. In our study too, the second most-affected site was the skin.

Pulmonary function tests can present restrictive or obstructive disorders in sarcoidosis, but generally, lung function is not greatly altered, as demonstrated in our population, which presented mean values of FVC, FEV_1_, and FEV_1_/FVC within normal ranges. However, 49% of the patients presented some change in these variables.^[29]^ We also found little change in lung function, as described in other studies.^[21]^ Echocardiographic signs of pulmonary hypertension were presented in only 3% of patients in our study, whereas other cohorts found a prevalence ranging from 5% to 28%.^[30–34]^

In 2009, the WASOG organized a task force to provide a better definition of clinical outcome status in sarcoidosis by examining patients 2 or 5 years after diagnosis.^[20]^ Authors disagreed on the ideal follow-up time for defining the disease as chronic. Neville et al^[14]^ and Judson et al^[35]^ used a 2-year cut-off point, while Baughman et al^[20]^ found more reliable results after 5 years of follow-up. Our patients were followed for at least 2 years after diagnosis.

Studies have already shown risk factors associated with poor sarcoidosis prognosis, e.g., Afro-American ethnicity,^[16,36–38]^ older age,^[16,21,37]^ changes in pulmonary function tests, and radiological stage 3.^[39]^ Pulmonary hypertension and pulmonary fibrosis are clinical features that have already been shown to be independent predictors of mortality among sarcoidosis patients.^[16,40,41]^ However, very few studies have developed scores, including multiple variables to define prognosis.

Walsh et al^[42]^ constructed an algorithm combining pulmonary function tests using the composite physiologic index (CPI) with computed tomography evaluation of the presence and extent of fibrosis, and the ratio of the main pulmonary artery diameter to the ascending aorta diameter, to categorize cases as good or poor prognosis, with consideration to mortality as the primary outcome.^[43]^ The study included 251 patients from a single center over a 10-year period and concluded that this score was a better predictor of mortality than any individual variable alone.

In another study, Drent et al^[44]^ showed that a score of radiological tomographic findings (thickening of the bronchovascular bundle, parenchymal consolidation, intra-parenchymal nodules, septal and nonseptal lines, focal pleural thickening, and enlargement of the lymph nodes) was related to lung function, i.e., a higher total score predicted respiratory functional impairment and a worse prognosis.^[45]^

The gender, age, and physiology (GAP) index, initially created as a predictor of mortality in idiopathic pulmonary fibrosis, has also been evaluated in sarcoidosis, and was shown to be related to a higher risk of death in stages 2 or 3, with age as the most important variable.^[16,46]^

Since mortality in sarcoidosis is usually low, particularly outside reference centers, we decided to establish a scoring system to assess prognosis, using COS instead of mortality, similar to that reported by Walsh et al.^[42]^ Using this system, we were able to predict patients who would develop chronic disease based on 3 clinical variables (respiratory symptoms, rheumatological markers, and pulmonary function tests).

The limitations of the study include the small number of patients; sample derived from a single center; a follow-up time of only 2 years; and the uniqueness of our sample. All patients were under clinical follow-up at a university hospital where cases of greater complexity are treated, usually using second-line medications, due to previous therapeutic failure.

## 5 Conclusion

Based on a simple algorithm derived from clinical, laboratory, and lung functional data, we were able to estimate the probability of the clinical evolution of patients with sarcoidosis, thus facilitating their management, which often requires more than 1 drug for treatment, and multiple follow-ups at reference centers.

## Author contributions

Study conception and design: MCL, TPA, MRA, RR; Acquisition of data: MCL, CHC, BRAS, EJCB, LPB, RR; Analysis and interpretation of data: MCL, TAP, MRA, RR; Drafting of manuscript: MCL, TAP, MRA, RR; Critical revision: MCL, TAP, MRA, RR.
